# Ambient particulate matter and microRNAs in extracellular vesicles: a pilot study of older individuals

**DOI:** 10.1186/s12989-016-0121-0

**Published:** 2016-03-08

**Authors:** Rodosthenis S. Rodosthenous, Brent A. Coull, Quan Lu, Pantel S. Vokonas, Joel D. Schwartz, Andrea A. Baccarelli

**Affiliations:** 1 Department of Environmental Health, Harvard T.H. Chan School of Public Health, Boston, MA USA; 2 VA Boston Healthcare System, Boston, MA USA

**Keywords:** Air pollution, Particulate matter, Cardiovascular disease, Extracellular vesicles, MicroRNAs

## Abstract

**Background:**

Air pollution from particulate matter (PM) has been linked to cardiovascular morbidity and mortality; however the underlying biological mechanisms remain to be uncovered. Gene regulation by microRNAs (miRNAs) that are transferred between cells by extracellular vesicles (EVs) may play an important role in PM-induced cardiovascular risk. This study sought to determine if ambient PM_2.5_ levels are associated with expression of EV-encapsulated miRNAs (evmiRNAs), and to investigate the participation of such evmiRNAs in pathways related to cardiovascular disease (CVD).

**Methods:**

We estimated the short- (1-day), intermediate- (1-week and 1-month) and long-term (3-month, 6-month, and 1-year) moving averages of ambient PM_2.5_ levels at participants’ addresses using a validated hybrid spatio-temporal land-use regression model. We collected 42 serum samples from 22 randomly selected participants in the Normative Aging Study cohort and screened for 800 miRNAs using the NanoString nCounter® platform. Mixed effects regression models, adjusted for potential confounders were used to assess the association between ambient PM_2.5_ levels and evmiRNAs. All *p*-values were adjusted for multiple comparisons. *In-silico* Ingenuity Pathway Analysis (IPA) was performed to identify biological pathways that are regulated by PM-associated evmiRNAs.

**Results:**

We found a significant association between long-term ambient PM_2.5_ exposures and levels of multiple evmiRNAs circulating in serum. In the 6-month window, ambient PM_2.5_ exposures were associated with increased levels of miR-126-3p (0.74 ± 0.21; *p* = 0.02), miR-19b-3p (0.52 ± 0.15; *p* = 0.02), miR-93-5p (0.78 ± 0.22; *p* = 0.02), miR-223-3p (0.74 ± 0.22; *p* = 0.02), and miR-142-3p (0.81 ± 0.21; *p* = 0.03). Similarly, in the 1-year window, ambient PM_2.5_ levels were associated with increased levels of miR-23a-3p (0.83 ± 0.23; *p* = 0.02), miR-150-5p (0.90 ± 0.24; *p* = 0.02), miR-15a-5p (0.70 ± 0.21; *p* = 0.02), miR-191-5p (1.20 ± 0.35; *p* = 0.02), and let-7a-5p (1.42 ± 0.39; *p* = 0.02). *In silico* pathway analysis on PM_2.5_-associated evmiRNAs identified several key CVD-related pathways including oxidative stress, inflammation, and atherosclerosis.

**Conclusions:**

We found an association between long-term ambient PM_2.5_ levels and increased levels of evmiRNAs circulating in serum. Further observational studies are warranted to confirm and extend these important findings in larger and more diverse populations, and experimental studies are needed to elucidate the exact roles of evmiRNAs in PM-induced CVD.

**Electronic supplementary material:**

The online version of this article (doi:10.1186/s12989-016-0121-0) contains supplementary material, which is available to authorized users.

## Background

Cardiovascular disease (CVD) is a leading cause of mortality and morbidity worldwide. In the United States (U.S.) alone, 31 % (2.5 million) of all deaths are attributed to CVD, and the proportion is expected to increase to ~40 % by 2030. The total health care cost of CVD was estimated to be ~580 billion dollars for the year 2012, but is projected to peak at ~1.2 trillion dollars by 2030 [1]. Among the risk factors for CVD, air pollution confers considerable risk. In fact, ambient air pollution has been estimated to account for ~3.7 million global deaths annually, and 80 % of those deaths have been attributed to CVD [2]. Associations of short- and long-term exposures to air particulate matter (PM) with increased CVD mortality and morbidity are, indeed, well documented [3, 4]. Despite significant past and recent efforts to reduce emissions, PM_2.5_ is still associated with CVD, even at the current levels [5]. Elucidating the subclinical biological changes that precede the onset of CVD, and are associated with air pollution, will help us to identify susceptible populations and inform new preventive policies to promote public health.

Particulate matter with an aerodynamic diameter of 2.5 μm or less (PM_2.5_) can penetrate deep into the lung, deposit in the alveolar area, and locally trigger oxidative stress and inflammatory responses. Epidemiological studies have consistently shown a substantial increase (3–76 %) in cardiovascular mortality in response to particulate matter exposures [3]. Experimental studies have revealed that compromised vascular and cardiac functions such as systemic inflammation [6], oxidative stress [7], and heart rate variability [8] due to prolonged exposure to PM_2.5_, precede serious CVD complications that often lead to death. Few biological mechanisms have been proposed to play a role in mediating the effects of PM_2.5_ from the lung to the cardiovascular system. One such mechanism is systemic inflammation, as several inflammatory markers, including C-reactive protein (CRP) [9], interleukin (IL)-6 [10], fibrinogen [11], and platelet activation [12] were found to be elevated in the blood in response to PM exposures. However, the mechanisms through which PM_2.5_ inhalation leads to CVD remain to be fully understood.

Extracellular vesicles (EVs) are small (40–1000 nm in diameter) double-lipid membrane vesicles [13] that play an important role in the cell-to-cell communication process [14]. EVs encapsulate and transfer biologically active molecules such as proteins and RNA molecules. Once EVs are internalized by the recipient cell(s), their cargo is released to the cytosol and could become functional [15]. microRNAs (miRNAs), small non-coding RNAs (~22 nucleotides long) [16] are abundantly present in EVs [13]. Because of their ability to degrade and/or suppress the translation of multiple mRNA molecules, miRNAs can post-transcriptionally regulate gene expression in cells [17]. In a systematic review, Vrijens et al. summarized data from recent human studies showing that miRNA levels change in response to air pollution exposures [18]. Izzoti et al., reviewed evidence suggesting that dysregulation of miRNA expression levels in response to persistent environmental exposures occurs primarily due to their interaction with components of the miRNA machinery such as the Drosha/DGCR8 processing complex, DICER and RNA-induced silencing complex (RISC) [19]. However, whether the levels of evmiRNAs in blood circulation are sensitive to environmental exposures, including ambient PM_2.5_ is still largely undetermined.

The objective of our study was to determine whether short-, intermediate-, and long-term exposures to ambient PM_2.5_ are associated with the levels of evmiRNAs detected in the blood of older individuals. We screened for 800 miRNAs in 42 serum samples of 22 randomly selected participants from the ongoing prospective Normative Aging Study (NAS). Additionally, we conducted pathway analysis to explore whether PM_2.5_-associated evmiRNAs regulate in CVD-related biological pathways. The findings of this study offer new avenues to explore the link between air pollution and CVD.

## Methods

### Sample selection

The U.S. Department of Veterans Affairs (VA) Normative Aging Study is an ongoing longitudinal study established in 1963, which enrolled 2,135 male volunteers, aged 21 to 80 years of age at enrollment from the Greater Boston area. All participants were free of any known chronic medical conditions at baseline. Further details of the study have been described previously [20]. Participants were invited to undergo comprehensive medical examinations every 3 to 5 years. By 2000, when satellite air pollution data became available, 749 of the original participants were still attending regular examinations. In this pilot study, we randomly selected 22 participants with available serum samples between the years 2000 and 2008. One serum sample from each participant at first visit (*n* = 22), and two additional samples for 10 out of 22 participants, from the following two visits (*n* = 20), were selected. In total, we analyzed 42 serum samples. The NAS was approved by the Institutional Review Boards of all participating institutions (#14027-102, reviewed on March 20, 2012). All participants provided written informed consent in accordance with the Declaration of Helsinki of ethical principles for medical research.

### Exposure assessment

A validated spatio-temporal land-use regression model was used to estimate the ambient moving-average PM_2.5_ levels at each participant’s home address for all time windows (1 day, 1 week, 1 month, 3 months, 6 months, and 1 year) preceding each blood withdrawal. We used our recently developed hybrid approach [21], which combines satellite data on aerosol optical density (AOD) with land-use variables, together with weather data and PM_2.5_ point- and area-source emissions from 78 monitoring stations across the study area from the U.S. Environmental Protection Agency (EPA) – National Emission Inventory [22]. We validated and successfully used these estimates in several recent investigations [23, 24]. In brief, this model uniquely integrates satellite AOD data, which are a measure of light scattering due to the presence of aerosols in the column of air spanning from Earth’s surface to the satellite. To estimate the levels of PM_2.5_ near the surface, we fitted a hybrid model using AOD and classic land-use regression data (i.e., elevation, distance to major roads, % open space, point emissions, and area emissions) together with several other meteorological variables such as temperature, wind speed, relative humidity, visibility, and height of the planetary boundary layer (PBL). Additional information on the development of this model is available elsewhere [25]. The model includes interaction terms with the PBL height to help capture differences in the fraction of particles that are near the ground and random slopes for each day to capture day-to-day changes in particle size and color. We also estimated daily PM_2.5_ concentration levels for all grid cells in the study domain for cells/days when AOD data were unavailable (e.g., cells covered by clouds) using grid-cell specific regressions against nearby monitors, land-use terms, and spatial smoothing. A final stage examined the difference of each daily PM_2.5_ observation on a 10 × 10-km grid cell mean. Differences were regressed against land-use terms within 100 m of the monitor, meteorology, and their interactions. This allows us to resolve the address-specific exposure. Ten-fold cross-validation showed very good model predictions with an out of sample R^2^ of 0.85 for daily PM_2.5_ measures.

### Blood collection, EVs isolation, and miRNA extraction

Peripheral blood was collected at each visit in EDTA tubes and centrifuged at 1500 × g for 15 min to separate the serum fraction following standard operating procedure. Aliquots of cell-free serum were stored immediately at −80 °C and thawed just before use for this study. For the isolation of EVs and miRNA extraction we used the ultracentrifugation method as first described by Théry et al. [26] with some modifications [27]. In brief, serum samples (~1.5 mL) were thawed on ice and centrifuged at 1000, 2000, and 3000 x g for 15 min at 4 °C, consecutively, to remove any remaining cell debris and large aggregates. Supernatants were then filtered using a 0.8-μm membrane unit (Millipore Corp., Bedford, MA) and further centrifuged using a TLA-110 fixed-angle rotor (Beckman Coulter, Danvers, MA) at 110,000 x g (*k* factor of 13) for 2 h at 4 °C. Isolated EVs were visualized by transmission electron microscopy and immune-gold labeling using antibodies for the CD-63 and CD-81 surface markers as described by Théry et al. [26]. (Additional file 1: Figure S1). Finally, miRNAs were extracted from the collected EVs using the miRNeasy Mini Kit (Qiagen, Valencia, CA) according to manufacturer’s instructions, and the RNA eluate was concentrated for downstream analysis using a vacuum concentrator.

### miRNA profiling

The Nanostring nCounter® platform was used to screen for expression level of 800 miRNAs. A volume of three microliters (3 μL) for each sample was prepared and analyzed according to the manufacturer’s protocol (NanoString Technologies, Seattle, WA). Briefly, a thermally controlled multiplexed ligation reaction was used to add specific DNA tag sequences on mature miRNAs. Following ligation, the excess tags were removed by affinity and the purified material was hybridized overnight at 65 °C with the nCounter® Human (V2) miRNA Expression Assay CodeSet. The nCounter® Prep Station was used to purify the hybridized probes and to attach the purified biotinylated complexes on the streptavidin-coated slides. miRNA counts were measured in two batches by the nCounter® Digital Analyzer. All samples were analyzed at NanoString’s laboratory (NanoString Technologies, Seattle, WA). The nSolver software (http://www.nanostring.com/products/nSolver) was used to analyze and normalize the raw data using the top 100 most abundant miRNAs in all samples, according to the manufacturer’s instructions. Positive controls were included to normalize for any differences in preparation, hybridization, and processing efficiency. Data were further tested for batch effects, normalized to the starting median serum volume and corrected for background noise using negative controls (internal probes and biological blank). Normalized miRNA counts were used for further analysis.

### Statistical analysis

Standard descriptive statistics were used to explore the characteristics of the study participants and the levels of evmiRNAs circulating in serum [reported as mean ± standard deviation (SD)]. We log-transformed (log_2_) the evmiRNA data to improve normality in the residuals. Univariate analysis was conducted between all PM_2.5_ moving averages (1-day, 1-week, 1-month, 3-month, 6-month, and 1-year) and all detected evmiRNAs. To maximize the completeness of our data set in the multivariate analysis, we included only the evmiRNAs that were detected in >90 % of our samples. For our analysis on the association of PM_2.5_ on the levels of evmiRNAs, we adjusted for other covariates such as age, body mass index (BMI), pack-years of smoking, total miRNA counts, and the numbers of red blood cells (RBCs), white blood cells (WBCs), and platelets. Additional covariates such as seasonality and years of education were also evaluated in our preliminary analysis; however, none of them improved the performance of our statistical models or significantly changed our findings; thus, we decided to use a more parsimonious model. The same covariates were used to explore the association between evmiRNAs and coronary heart disease (CHD) history (yes/no) in the study participants, using logistic regression models. To incorporate all data from the repeated measures of each of the detected evmiRNAs in our study population, the mixed effects models approach with random intercept for each participant was used. The Benjamini and Hochberg (BH) procedure was used to control for multiple comparisons (*n* = 186, 31 miRNAs x 6 time windows) and False Discovery Rate (FDR) [28]. A two-sided BH FDR of <0.05 was considered significant. Lastly, Spearman’s correlation analysis was conducted to explore the longitudinal correlations of the evmiRNAs in repeatedly collected serum samples. All statistical analyses were conducted using SAS version 9.4 (SAS Institute Inc., Cary, NC).

### miRNA targets and biological network analysis

We used the Ingenuity Pathway Analysis (IPA) software (Ingenuity Systems®, Redwood City, CA) to identify miRNA putative targets and explore downstream biological networks. Only evmiRNAs that were found to be associated with ambient PM_2.5_ levels were included in this analysis. The miRNA Target Filter tool, which links predicted and experimentally validated mRNA targets to each miRNA from TarBase, miRecords and TargetScan, was used. In the pathway and biological network analysis, we used the following parameters: (a) Confidence level: Experimentally Observed, (b) Species: Human and (c) Biofluids: Blood and Serum/Plasma. Further *in silico* analysis was conducted to explore the biological relevance of PM_2.5_-associated evmiRNAs and signaling pathways related to the cardiovascular system and inflammatory responses, that we selected a priori.

## Results

### Characteristics of study participants

In this study, all participants (*n* = 22) were non-hispanic white men, with a mean age of 75 ± 6.6 years, BMI of 26.8 ± 2.9 kg/m^2^, RBC counts of 4.7 ± 0.4 thousands/mm^3^, WBC counts of 6.5 ± 2.5 thousands/mm^3^, and platelet counts of 231.9 ± 51.8 thousands/mm^3^, at the first examination (Additional file 2: Table S1). Sixteen participants (62.8 %) were former smokers and six (37.2 %) never smoked. Former smokers quitted smoking at least 12 years prior to enrollment and reported a mean of 15.8 ± 15.2 pack-years of smoking. Seven participants (31.8 %) had a history of CHD at baseline. The ambient PM_2.5_ levels (mean ± SD) for 1-day, 1-week, 1-month, 3-month, 6-month, and 1-year moving averages were estimated to be 10.76 ± 6.85 μg/m^3^, 10.48 ± 2.81 μg/m^3^, 10.32 ± 2.36 μg/m^3^, 11.05 ± 1.78 μg/m^3^, 11.14 ± 1.14 μg/m^3^, and 11.14 ± 0.97 μg/m^3^, respectively.

### Association of ambient PM_2.5_ levels and evmiRNAs in serum

**Fig. 1:**
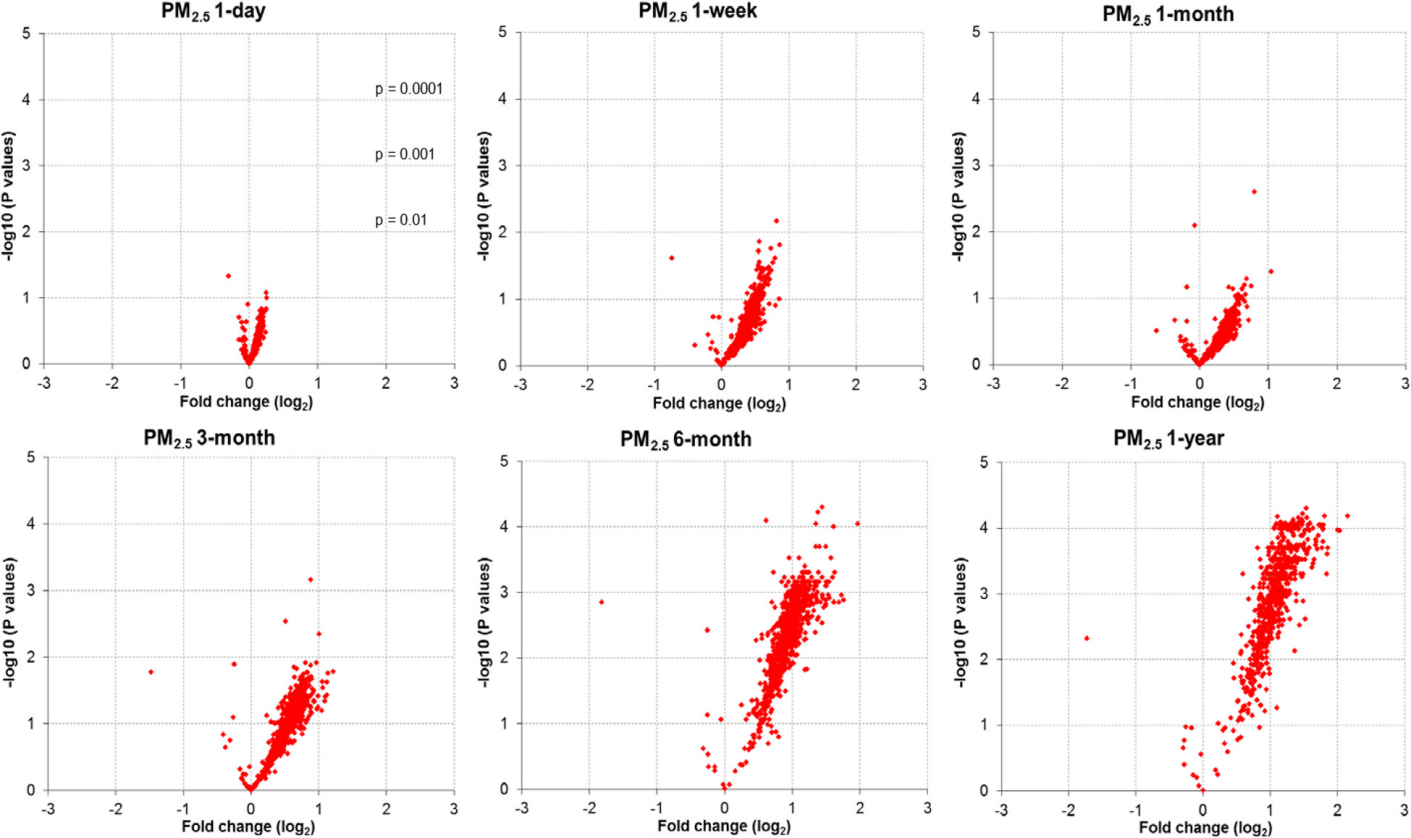
Univariate association of ambient PM_2.5_ levels and all measured miRNAs (*n* = 798) in extracellular vesicles (EVs). PM_2.5_ 1-day, 1-week, 1-month, 3-month, 6-month, and 1-year indicate the ambient PM_2.5_ moving average levels 1 day, 1 week, 1 month, 3 months, 6 months, and 1 year before blood sample collection; −log_10_ (*P* values) indicates log_10_ transformed *p*-values of the fold-change association of PM_2.5_ moving average time windows on each of the detected miRNAs in serum extracellular vesicles; fold change (log_2_) indicates the log_2_ transformed fold change of each of the detected miRNAs in serum extracellular vesicles in response to PM_2.5_ moving average time windows

**Fig. 2:**
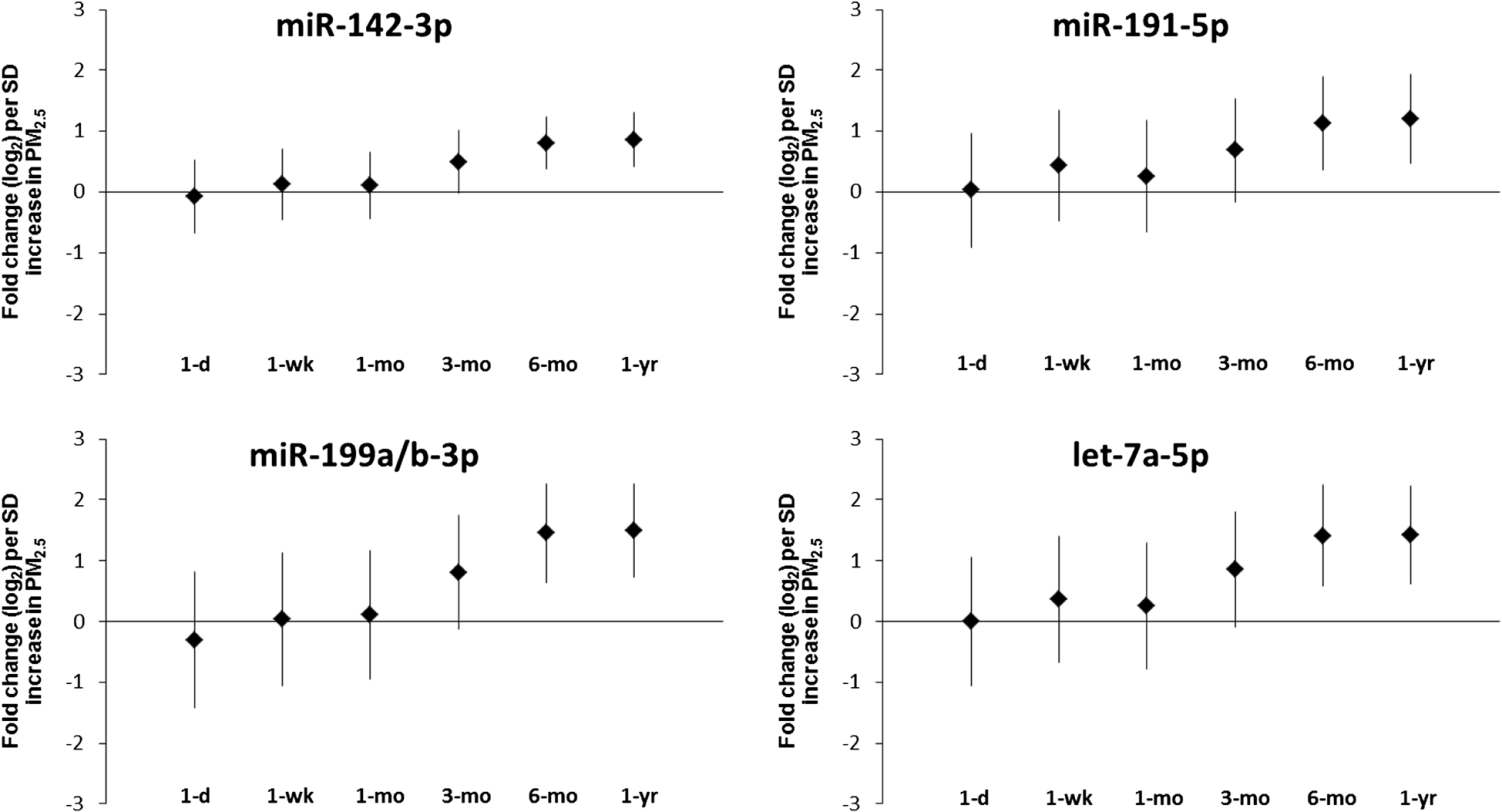
Fold change (95 % CI) of selected miRNAs in extracellular vesicles over different time windows of ambient PM_2.5_ levels. Fold changes (95 % CI) for miR-142-3p, miR-191-5p, miR-199a/b-3p, and let-7a-5p in response to ambient PM_2.5_ 1-day, 1-week, 1-month, 3-month, 6-month, and 1-year moving averages before blood sample collection, respectively; all estimates were adjusted for age; body mass index (BMI); number of pack-years of smoking; total miRNA counts, and the number of red blood cells (RBCs), white blood cells (WBCs), and platelets; SD indicates standard deviation

We determined the association between ambient PM_2.5_ exposures and levels of evmiRNAs circulating in serum using short- (1-day), intermediate- (1-week and 1-month), and long-term (3-month, 6-month, and 1-year) PM_2.5_ moving averages. The volcano plots depict the univariate association (fold change) per SD increase of PM_2.5_ on all evmiRNAs detected in at least one sample (*n* = 798), at all tested PM_2.5_ moving averages (Fig. 1; Additional file 3: Table S2). Of the 798 detected evmiRNAs, 31 were present in >90 % of all analyzed serum samples and thus selected for further analysis. In Table 1, we show the levels and number of serum samples in which each of the 31 evmiRNAs was detected. Mixed effects regression models, adjusted for age, BMI, pack-years of smoking, total miRNA counts, and counts of RBCs, WBCs, and platelets, revealed a statistically significant long-term (6-month and 1-year) association of ambient PM_2.5_ exposures and levels of evmiRNAs circulating in serum. All results were adjusted for multiple comparisons using FDR correction. Of the 31 tested evmiRNAs, we found that 16 were statistically significantly associated with either the 6-month or 1-year PM_2.5_ moving averages. We herein show the top-5 evmiRNAs (by *p-*value) for each PM_2.5_ moving average time window and their fold-change levels in response to every PM_2.5_ SD increment (Table 2). We found the most significant associations between 6-month PM_2.5_ moving average and fold change of miR-126-3p (0.74 ± 0.21; *p* = 0.02), miR-19b-3p (0.52 ± 0.15; *p* = 0.02), miR-93-5p (0.78 ± 0.22; *p* = 0.02), miR-223-3p (0.74 ± 0.22; *p* = 0.02), and miR-142-3p (0.81 ± 0.21; *p* = 0.03). Furthermore, for the 1-year PM_2.5_ moving average, miR-23a-3p (0.83 ± 0.23; *p* = 0.02), miR-150-5p (0.90 ± 0.24; *p* = 0.02), miR-15a-5p (0.70 ± 0.21; *p* = 0.02), miR-191-5p (1.20 ± 0.35; *p* = 0.02), and let-7a-5p (1.42 ± 0.39; *p* = 0.02) showed the most significant associations. The complete list of all evmiRNAs their fold changes in response to short-, intermediate-, and long-term PM_2.5_ moving averages are shown in Additional file 3: Table S2. In Fig. 2, we show the fold change (95 % CI) of four selected evmiRNAs (miR-142-3p, miR-191-5p, miR-199a/b-3p and let-7a-5p), in response to ambient PM_2.5_ levels at different time windows. These evmiRNAs showed the highest statistical significance in both long-term (6-month and 1-year) PM_2.5_ time windows, as well as the largest overall fold change. Additionally, we provide the fold change (95 % CI) of other evmiRNAs (let-7 g-5p, miR-126-3p, miR-15a-5p, miR-223-3p, miR-23a-3p and miR-93-5p) that were significantly associated with both long-term PM_2.5_ time windows (6-month and 1-year), but were associated with lower overall fold change (Additional file 4: Figure S2). Finally, we show the longitudinal correlations of all 31 evmiRNAs detected in serum samples from three concecutive visits of 10 participants (*n* = 30) over the sampling period 2000–2008 (Table 3). This analysis revealed a weak correlation of the detected evmiRNAs over the period of time that samples were collected.

**Table 1:** Levels of miRNAs in extracellular vesicles detected in >90 % of analyzed serum samples (*n* = 42)

miRNA	n (obs)^a^	Mean (SD)^b^
let-7a-5p	41	6.25 (2.60)
let-7b-5p	41	5.23 (1.88)
let-7 g-5p	42	8.24 (1.20)
miR-106b-5p	41	5.16 (1.35)
miR-1246	41	2.55 (3.87)
miR-126-3p	42	8.16 (1.41)
miR-130a-3p	42	5.72 (1.02)
miR-142-3p	42	8.68 (1.36)
miR-144-3p	42	8.83 (1.25)
miR-146a-5p	41	5.15 (2.12)
miR-150-5p	42	7.85 (1.86)
miR-15a-5p	42	7.44 (1.38)
miR-15b-5p	42	7.27 (1.24)
miR-16-5p	42	8.24 (1.57)
miR-181a-5p	38	4.22 (1.96)
miR-185-5p	42	6.05 (0.71)
miR-191-5p	41	6.89 (2.19)
miR-199a/b-3p	40	5.52 (2.61)
miR-19b-3p	41	6.27 (1.56)
miR-20a/b-5p	39	5.16 (2.07)
miR-223-3p	42	9.70 (1.43)
miR-23a-3p	42	7.30 (1.52)
miR-25-3p	42	8.53 (1.10)
miR-30d-5p	41	4.82 (1.11)
miR-320e	42	7.45 (0.77)
miR-342-3p	39	4.29 (1.47)
miR-4454	42	8.69 (1.28)
miR-451a	42	12.64 (1.07)
miR-505-3p	41	3.24 (1.64)
miR-720	42	6.18 (1.41)
miR-93-5p	41	6.30 (1.81)

^a^Number of serum samples in which each miRNA was detected

^b^Expressed as log_2_

**Table 2:** Associations between ambient PM_2.5_ moving average time windows and levels of miRNAs in extracellular vesicles (evmiRNAs)

Exposure window	miRNA	Fold change^a^	SE	FDR adjusted
				*P* value
1-day				
	miR-30d-5p	−0.56	0.17	0.03
	miR-25-3p	−0.30	0.14	0.16
	miR-342-3p	−0.44	0.22	0.20
	miR-106b-5p	−0.56	0.29	0.23
	miR-451a	−0.27	0.16	0.30
1-week				
	miR-451a	−0.33	0.15	0.16
	miR-25-3p	−0.21	0.14	0.37
	miR-720	−0.33	0.25	0.44
	miR-4454	−0.29	0.22	0.44
	miR-30d-5p	−0.23	0.20	0.53
1-month				
	miR-451a	−0.24	0.15	0.30
	miR-19b-3p	0.17	0.18	0.65
	miR-146a-5p	0.27	0.40	0.78
	miR-150-5p	0.23	0.32	0.78
	miR-1246	−0.43	0.70	0.80
3-month				
	miR-19b-3p	0.47	0.17	0.05
	miR-93-5p	0.57	0.25	0.12
	miR-150-5p	0.64	0.28	0.13
	miR-1246	−1.56	0.73	0.17
	miR-142-3p	0.50	0.26	0.22
6-month				
	miR-126-3p	0.74	0.21	0.02
	miR-19b-3p	0.52	0.15	0.02
	miR-93-5p	0.78	0.22	0.02
	miR-223-3p	0.74	0.22	0.02
	miR-142-3p	0.81	0.21	0.03
1-year				
	miR-23a-3p	0.83	0.23	0.02
	miR-150-5p	0.90	0.24	0.02
	miR-15a-5p	0.70	0.21	0.02
	miR-191-5p	1.20	0.35	0.02
	let-7a-5p	1.42	0.39	0.02

^a^Estimated fold change (log_2_) of the levels of evmiRNAs per standard deviation (SD) increase in ambient PM_2.5_ levels for every time window; all models were adjusted for age, body mass index (BMI), pack-years of smoking, total miRNA counts, and the number of red blood cells (RBCs), white blood cells (WBCs), and platelets; SE indicates standard error

**Table 3:** Within-individual Spearman’s correlations of miRNAs in serum extracellular vesicles from each visit

miRNA	1^st^ vs. 2^nd^	2^nd^ vs. 3^rd^	1^st^ vs. 3^rd^
hsa-let-7a-5p	−0.49	0.12	0.36
hsa-let-7b-5p	−0.75	0.32	0.12
hsa-let-7 g-5p	0.01	0.43	0.35
hsa-miR-106b-5p	−0.03	0.14	0.18
hsa-miR-1246	−0.21	0.78	−0.13
hsa-miR-126-3p	−0.24	0.07	0.38
hsa-miR-130a-3p	−0.18	0.30	−0.10
hsa-miR-142-3p	−0.26	0.38	−0.04
hsa-miR-144-3p	−0.21	0.58	−0.05
hsa-miR-146a-5p	−0.52	−0.13	0.07
hsa-miR-150-5p	−0.33	0.38	0.15
hsa-miR-15a-5p	−0.35	0.62	−0.42
hsa-miR-15b-5p	−0.12	0.68	0.12
hsa-miR-16-5p	0.42	0.36	0.58
hsa-miR-181a-5p	−0.58	−0.21	−0.28
hsa-miR-185-5p	0.38	0.50	0.10
hsa-miR-191-5p	−0.81	0.43	−0.67
hsa-miR-199a/b-3p	−0.59	0.64	−0.32
hsa-miR-19b-3p	0.16	0.37	−0.30
hsa-miR-20a/b-5p	−0.36	0.24	0.31
hsa-miR-223-3p	−0.24	0.24	−0.50
hsa-miR-23a-3p	−0.48	0.52	−0.41
hsa-miR-25-3p	0.36	0.21	0.10
hsa-miR-30d-5p	0.16	0.15	0.09
hsa-miR-320e	−0.01	−0.36	−0.13
hsa-miR-342-3p	0.12	0.30	0.50
hsa-miR-4454	−0.07	0.65	0.41
hsa-miR-451a	0.70	0.56	0.14
hsa-miR-505-3p	−0.59	0.09	−0.32
hsa-miR-720	−0.37	0.42	0.15
hsa-miR-93-5p	−0.42	0.45	−0.26

### miRNA targets, pathway analysis, and biological relevance

**Fig. 3:**
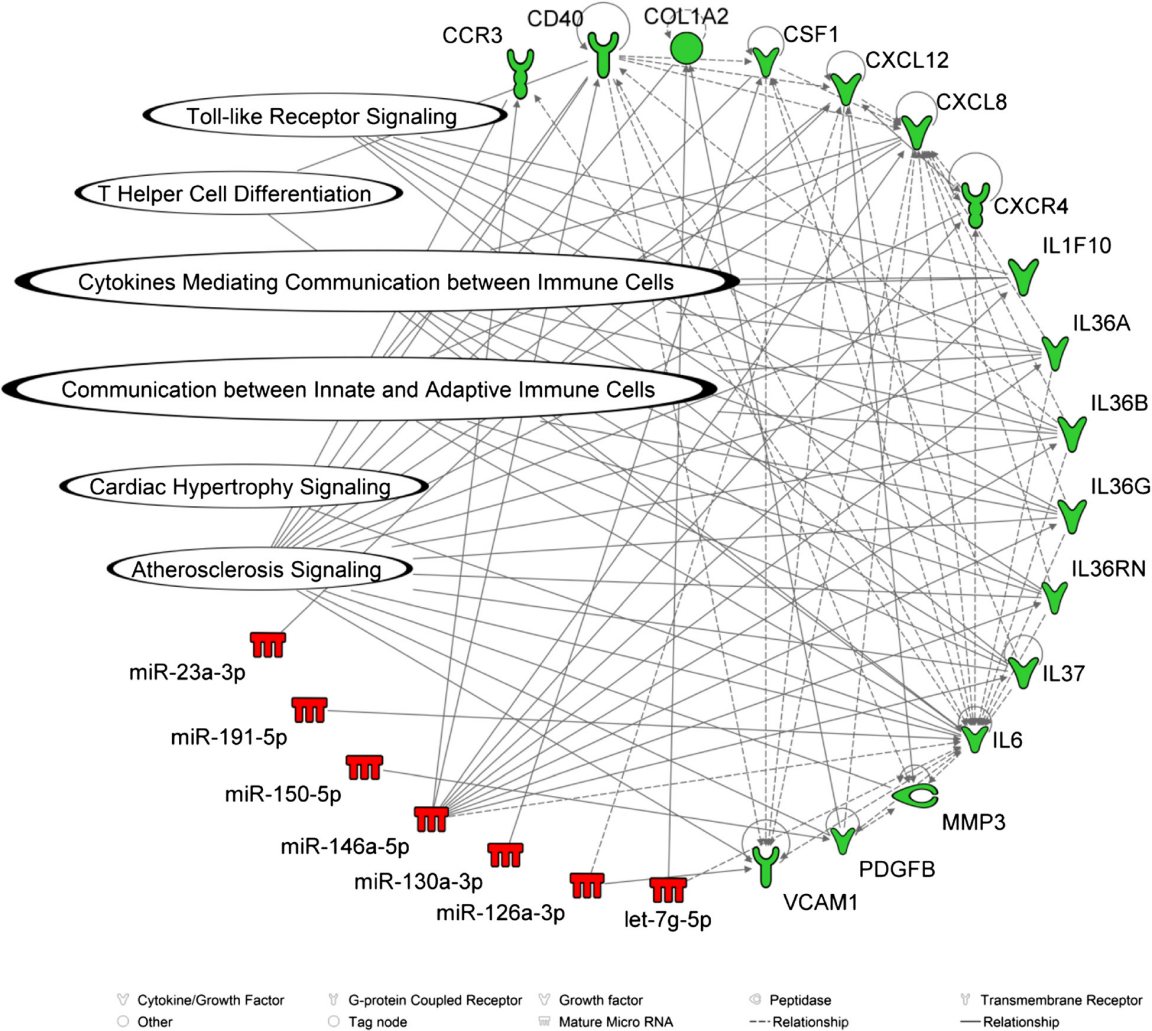
Pathway analysis illustrating the link between PM_2.5_-associated miRNAs in extracellular vesicles and biological pathways related to cardiovascular disease. Selected miRNAs (in red) associated with long-term ambient PM_2.5_ levels and their experimentally validated mRNA targets (in green) as identified by the Ingenuity Pathway Analysis software. The biological pathways involved in cardiovascular disease (CVD) for all miRNA-mRNA interactions are also shown here. Solid lines represent a direct interaction and dotted lines represent an indirect interaction

To explore the plausible biological function of miRNAs, detected in EVs, that were associated with ambient PM_2.5_ exposures, we included all evmiRNAs (*n* = 16) that reached statistical significance in the adjusted models (Additional file 5: Table S3) for further *in-silico* analysis using the IPA software. We found an interaction between seven evmiRNAs (let-7 g-5p, miR-126-3p, miR-130a-3p, miR-146a-5p, miR-150-5p, miR-191-5p, and miR-23a-3p) and 16 unique, experimentally validated mRNA targets (CCR3, CD40, COL1A2, CSF1, CXCL12, CXCL8, CXCR4, IL1F10, IL36A, IL36B, IL36G, IL36RN, IL37, IL6, PDGFB, and VCAM1). These interactions were enriched in significant cardiovascular-related pathways such as atherosclerosis, cardiac hypertrophy, and inflammatory responses (Fig. 3). When we further explored associations between all evmiRNAs and CHD history, we observed an odds ratio (95 % CI) of 2.32 (95 % CI: 1.33, 4.04; *p* = 0.05) for miR-4454, 2.24 (95 % CI: 1.34, 3.75; *p* = 0.07) for miR-720, 0.38 (95 % CI: 0.20, 0.74; *p* = 0.04) for miR-130a-3p, and 0.47 (95 % CI: 0.27, 0.81; *p* = 0.05) for miR-106b-5p. No significant associations were observed between other evmiRNAs and CHD after adjusting for FDR (Additional file 6: Figure S3; Additional file 7: Table S4). Odds ratios for the association between all evmiRNAs and CHD are shown in Additional file 6: Figure S3 and Additional file 7: Table S4.

## Discussion

In this study, we identified an association between 16 evmiRNAs and long-term ambient PM_2.5_ levels. To further investigate their biological function, we conducted *in silico* pathway analysis using the IPA software. We identified seven evmiRNAs (let-7 g-5p, miR-126-3p, miR-130a-3p, miR-146a-5p, miR-150-5p, miR-191-5p, and miR-23a-3p) with 16 experimentally validated mRNA targets (CCR3, CD40, COL1A2, CSF1, CXCL12, CXCL8, CXCR4, IL1F10, IL36A, IL36B, IL36G, IL36RN, IL37, IL6, PDGFB, and VCAM1) that are involved in several CVD-related signaling pathways such as atherosclerosis, cardiac hypertrophy, Toll-like receptor, T-Helper cell differentiation, cytokines mediating communication between immune cells, and inter-communication between innate and adaptive immune cells. Exploratory analysis on the association between levels of evmiRNAs and CHD history suggested that overall higher levels of evmiRNAs in the blood circulation were associated with no CHD history. Collectively, these results are consistent with our hypothesis that air pollution is associated with the levels of evmiRNAs circulating in blood, which may participate in biological functions relevant to CVD-related pathways.

Using our newly developed spatio-temporal model to estimate the levels of PM_2.5_, we found significant associations of the levels of certain evmiRNAs with relatively low differences in the concentrations of ambient PM_2.5_. A recent study by Madrigano et al. in the Worcester Heart Attack Study (Worcester, MA), reported a significant 16 % increase in the odds of acute myocardial infraction per interquartile range increase (0.59 μg/m^3^) of PM_2.5_ in a study with a similarly narrow range of 1-year PM_2.5_ exposures (10.44 ± 1.36 μg/m^3^) [5]. Taken together, these findings provide biological plausibility of the underlying molecular mechanisms in the association of PM_2.5_ exposures and CVD, even at lower concentrations and variation, such as those measured in the Greater Boston area.

The overall increase in evmiRNAs levels associated with PM_2.5_ exposures may have originated from either of both of two different biological mechanisms, i.e., an increase in the numbers of EVs released in the blood circulation by blood cells or tissues sensitive to air pollution, and/or an increase in the amount of specific miRNAs loaded in EVs prior to their release in the blood circulation. Bollati et al., showed a similar pattern of an overall increase in the levels of miRNAs detected in EVs after short-term exposure to PM_10_ in a group of foundry workers [27]. The investigators used an endogenous control to normalize their miRNA expression data which provided additional support to the hypothesis that the overall increase in the detected miRNA levels in response to air pollution might be due to an increase in the selective process of miRNAs loading in EVs, prior to their release in the blood circulation. However, they recognized that the possibility of an increase in the numbers of EVs could have also occurred. Interestingly, recent studies found that elevated numbers of EVs in healthy subjects were linked to increased risk for developing CVD [29, 30]. Future research is warranted to determine to which extent the overall increase in evmiRNA levels observed in response to PM_2.5_ is attributed to increased EV numbers and/or increased EVs miRNA load, prior to their release.

Our findings are consistent with previous in vitro and in vivo studies showing that miRNA expression in blood cells or other target tissues is sensitive to air pollutants. For instance, in some of our previous work, we demonstrated the differential expression of miR-126-3p, miR-146a-5p, and hsa-let-7 g in blood leukocytes in response to short-term PM exposures [31–33]. In addition, a recent in vivo study in rats demonstrated a dose–response relationship between exposure to PM and miRNA expression levels in the left ventricle of the heart; among others, miR-19b-3p, miR-146a-5p, miR-150-5p and miR-191-5p were differentially expressed in response to higher doses of PM [34]. Furthermore, Fry and colleagues showed that miR-199a/b-3p and miR-223-3p were upregulated in sputum cells in response to air pollution [35]. In agreement with these studies, our data show an increase in the levels of miR-126-3p, miR-146a-5p, miR-150-5p, miR-19b-3p, miR-191-5p, miR-199a/b-3p, miR-223-3p, and let-7 g-5p in EVs circulating in serum. Further, we herein provide data on several other evmiRNAs sensitive to PM_2.5_ (i.e., miR-142-3p, miR-23a-3p, and miR-93-5p) that have not been reported previously. Additional analysis of the within-individuals long-term correlation of evmiRNAs showed an overall weak correlation. This finding suggests that PM_2.5_-associated evmiRNAs may be more sensitive to between-individual, rather than within-individual, factors and that their change in response to air pollution does not persist in the long-term. For example, Giusti et al. showed that EV release in response to cellular stimulation by fetal bovine serum (FBS) is a time-dependent process in which several EV parameters, including number and composition, are altered [36]. Furthermore, Tian et al. revealed that the uptake of EVs by target cells is a very dynamic and fast process [37]. Thus, we hypothesize that the EVs cargo (i.e., miRNAs) is associated to this dynamic change as well. Collectively, these studies provide evidence in support of our findings for weak long-term correlations between evmiRNAs.

To gain more insights about the mRNA target(s) of each of the PM-associated evmiRNAs and their plausible impact on cellular processes and downstream biological pathways, we utilized the IPA databases. We restricted our analysis to only those miRNA-mRNA interactions that were experimentally validated by either in vitro or in vivo studies. Of the 16 evmiRNAs included in the analysis, seven exhibited biological functional interactions with 16 unique mRNAs enriched in CVD-related pathways such as atherosclerosis, inflammation, and cytokine-mediated pathways. For example, this analysis revealed miRNA-mRNA interactions between miR-146a-5p and critical pro-inflammatory cytokines such as IL-6 and the IL-36 family (IL-36A, IL-36B and IL-36G). miR-146a-5p is an important regulator of IL-6 via the nuclear factor-kappa-B (NF-kB) pathway, and can regulate the expression of NF-kB in macrophages by targeting the TRAF6 and IRAK1 proteins upstream the NF-kB signaling pathway [38]. In turn, IL-6 was found to regulate the expression of the miR-17/92 cluster, including miR-19b-3p, via the activation of STAT3, which can directly target BMPR2, a surface protein receptor that is expressed in endothelial and vascular smooth muscle cells [39]. The IL-36 family is released by either activated immune cells or epithelial cells and contributes to inflammatory response via the NF-kB pathway [40], which makes IL-36 targeting a fine-tuner of the NF-kB pathway [41]. In addition, miR-146a-5p targets TRAF6 and IRAK1, proteins that are part of the CD40 signaling pathway [42]; CD40, which serves an important role in cellular communication during inflammatory responses, is implicated in atherosclerosis [43]. IPA also revealed interactions of miR-146a-5p and miR-23a-3p with the chemokines C-X-C motif ligand 8 and 12 (CXCL8 and CXCL12), respectively. Both cytokines have been reported to regulate inflammation [44, 45], and thus their fine-tuning by miRNAs could be critical. Indeed, CXCL8 can be regulated by mir-146a-5p [46] and miR-20a-5p [47], whereas CXCL12 can be regulated by mir-23a-3p [48]. Further, we found an interaction between the endothelial-specific miR-126a-3p and vascular adhesion molecule 1 (VCAM-1), a trans-membrane receptor in endothelial cells. VCAM-1 is expressed by endothelial cells in response to inflammation and plays critical role in recruiting leukocytes at the site of inflammation [49]. A study by Harris et al., found that miR-126a-3p can regulate the expression of VCAM-1 in endothelial cells with implications for vascular inflammation and atherosclerosis [50]. In the same context, we found an interaction between miR-150-5p and the platelet-derived growth factor beta (PDGF-B), a protein that is expressed by smooth muscle endothelial and epithelial cells. PDGF-B plays a central role in cell proliferation and has been implicated in inflammatory responses and atherosclerosis. A study by Shen and colleagues found that miR-150-5p can directly target and regulate the expression of PDGF-B in retina epithelial cells [51]. Lastly, miR-126a-3p has also a regulatory role for the expression of CXCL12 via the inhibition of CXCR4 by RGS16, a signaling pathway that is involved in atherosclerosis and inflammation [52].

We further explored the association between evmiRNAs and coronary heart disease history in the study participants. We observed some signals of association between certain evmiRNAs and increased (miR-4454 and miR-720) or decreased (miR-106b-5p and miR-130a-3p) odds ratio of CHD history. Overall, PM_2.5_-induced evmiRNAs did not show significant associations with CHD history, except miR-130a-3p; however, we observed that higher levels of PM_2.5_-induced were more likely to be measured in participants with no CHD history. These findings suggest that an adaptive response to long-term PM_2.5_ exposures might be in place. Yamamoto et al., showed that the expression of miR-144-3p in peripheral blood was induced by diesel exposures, and that it was involved in downstream adaptive response pathways to oxidative stress triggered by air pollutants [53]. In our study, we found that miR-144-3p levels were marginally significantly induced (*p* = 0.06) by long-term PM_2.5_ exposures (Additional file 5: Table S3) and that higher levels were more likely to be observed in participants with no CHD history [OR: 0.49 (0.24, 0.98); *p* = 0.17] (Additional file 6: Figure S3; Additional file 7: Table S4). Our findings are limited by the sample size and use of cross-sectional data, and any inferences on the biological relevance of PM-induced evmiRNAs in the development or progression of CVD-related outcomes must be drawn very carefully. However, we provide data to encourage further research in larger prospective studies to confirm our findings, as well as experimental studies to determine the role of PM_2.5_-induced miRNAs in the development and progression of CVD.

Due to limited sample volume, we were not able to validate the miRNA data with a different platform such as real-time qPCR. However, Knutsen et al. showed an overall high correlation (r = 0.703–0.797) when they compared the miRNA fold-change values in NanoString nCounter® and other platforms, including real-time qPCR [54]. For the same reason, we could not quantify the number of EVs, nor we could characterize the different subpopulations (e.g., based on the tissue of origin) of EVs in the blood of the study participants. It is well documented that the number of circulating EVs in the blood varies between individuals, and that EVs sub-populations can originate from several tissues. In the blood, EVs primarily originate from blood cells (i.e., red blood cells, platelets, and white blood cells), and, to a lesser extent, from endothelial cells, lung epithelial cells, and cardiomyocytes [14]. To address these limitations, we controlled for different cell/tissue sources in the statistical analysis by adjusting for the number of RBCs, WBCs, and platelets. We also controlled for the between-individuals difference in the numbers of EVs by adjusting for the total number of miRNA counts measured in each sample.

We used ultracentrifugation to isolate EVs, which may not prevent contamination from other sources of circulating miRNAs in serum. Arroyo and colleagues showed that miRNAs bound to Argonaute2 (Ago2) protein represent an additional source of circulating miRNAs in blood [55]. Due to their different physical properties, these complexes are not expected to pellet with EVs, but remain in the supernatant fraction after ultracentrifugation. Nonetheless, aggregates of Ago2:miRNA complexes might co-precipitate with EVs in the pellet during isolation. To have some indication on possible contamination in our samples, we compared our miRNA data with the data generated by Arroyo et al. We found that 11 out of the 16 miRNAs that were significantly associated with long-term exposures to PM_2.5_ in our study, were also detected by Arroyo et al. in the EVs (pellet) fractions (let-7a-5p, miR-126-3p, miR-142-3p, miR-146a-5p, miR-150-5p, miR-191-5p, miR-19b-3p, miR-199a/b-3p, miR-23a-3p, miR-223-3p and miR-93-5p). This is particularly encouraging for the validity and efficiency of our method; however, we cannot completely exclude the possibility of minor contamination from co-precipitated Ago2:miRNA complexes. In fact, we identified one miRNA (let-7 g-5p) in our data that was found to be exclusively enriched in Ago2 fractions in the Arroyo study. Four miRNAs (miR-1246, miR-130a-3p, miR-15a-5p and miR-505-3p) were not reported in this study due to technical reasons. A recent study also compared miRNA profiles between cell-free serum, which includes both EVs and miRNA:protein complexes, and EVs isolated from serum by ultracentrifugation; among others, all of the 16 miRNAs that we found to be associated with long-term PM_2.5_ exposures, including miR-1246, miR-15a-5p, miR-130a-3p and miR-505-5p, were detected in EVs [56].

To further explore the origin of the evmiRNAs detected in our study, we searched for tissue-specific miRNA expression studies in the literature. Several miRNAs were found to be highly expressed in cells and tissues that contribute to the total population of EVs in the blood circulation. For example, studies have linked the expression of miR-126-3p to the heart and lung endothelial cells [57]; miR-142-3p to blood mononuclear cells and the lymphatic system [58]; miR-146a-5p to white blood cells and the respiratory system [59]; miR-150-5p to the heart, mononuclear blood cells, and the lymphatic system [58, 59]; miR-199a/b-3p to the heart and blood mononuclear cells [60, 61]; miR-19b-3p to the heart [34]; miR-223-3p to blood cells [58]; and miR-93-5p to heart endothelial cells [62]. Unfortunately, mRNA gene expression data on target tissues (e.g., heart and blood cells) that could provide additional information on the miRNA-mRNA interactions were not obtainable in vivo. To address this challenge, we restricted our *in silico* pathway analysis to data that were experimentally validated (in vitro or in vivo) and were relevant to the cardiovascular system. These findings support the hypothesis that specific miRNAs detected in EVs might be released in the blood circulation from cells/tissues sensitive to air pollution.

In our study, all participants were non-hispanic white older males with very similar socioeconomic status. We acknowledge that the characteristics of the study participants limit the generalizability of our findings to other populations; however, the homogeneity of the study participants helped to reduce potential confounding. This study has several other strengths, including the use of hybrid spatio-temporal land-use regression models to estimate the ambient PM_2.5_ levels at the residential address of study participants. Exposure assessment has been a major limitation in many studies; however, our newly developed validated hybrid approach integrates satellite AOD data, land-use variables, weather data, and PM_2.5_ source emissions data from the U.S. EPA to calculate the ambient PM_2.5_ levels. Thus, this method allows us to more accurately and reliably measure PM_2.5_ ambient levels at a 10 × 10-km resolution. Furthermore, we used a state-of-the-art technology such as the NanoString nCounter® platform to screen for 800 miRNAs. This provides us with the opportunity to screen for a large number of evmiRNAs that have not been investigated in relation to air pollution previously. Lastly, we utilized available data from validated questionnaires and medical records in the NAS including critical covariates considered in the analysis such as age, anthropometric measures, pack-years of smoking, and counts of blood cells.

## Conclusions

In summary, we showed that long-term ambient PM_2.5_ levels, is associated with the levels of several evmiRNAs circulating in the blood of older individuals. We found that several of these evmiRNAs are enriched in CVD-related pathways, which may have implications for the association between air pollution and CVD. Further research is warranted to replicate our findings in larger and more diverse populations, as well as determine the role of air pollution-induced evmiRNAs in human health.

## Additional files

Additional file 1: Figure S1. Morphological characterization of serum extracellular vesicles (EVs). Preparations of EVs were imaged by transmission electron microscopy (TEM). (a) non-labeled EVs, (b) EVs labeled with gold-conjugated anti-CD63 antibody, (c) EVs labeled with gold-conjugated anti-CD81 antibody. Images were taken by a JEOL 1200EX microscope coupled with an AMT 2 k CCD camera, at the Harvard Medical School Electron Microscopy Core. (DOCX 4627 kb)

Additional file 2: Table S1. Characteristics of the study participants at first examination (*n* = 22). (DOCX 18 kb)

Additional file 3: Table S2. Univariate association of ambient PM_2.5_ levels and all measured miRNAs (*n* = 798) in extracellular vesicles (EVs). (XLSX 289 kb)

Additional file 4: Figure S2. Fold change (95 % CI) of selected miRNAs in extracellular vesicles over different time windows of ambient PM_2.5_ levels. Fold changes (95 % CI) for let-7 g-5p, miR-126-3p, miR-15a-5p, miR-223-3p, miR-23a-3p and miR-93-5p in response to ambient PM_2.5_ one-day, one-week, one-month, three-month, six-month, and one-year moving averages before blood sample collection, respectively; all estimates were adjusted for age; body mass index (BMI); number of pack-years of smoking; total miRNA counts, and the number of red blood cells (RBCs), white blood cells (WBCs), and platelets; SD indicates standard deviation. (DOCX 58 kb)

Additional file 5: Table S3. Associations between ambient PM_2.5_ moving average time windows and levels of miRNAs in extracellular vesicles. (DOCX 31 kb)

Additional file 6: Figure S3. Forest plot showing odds ratios (95 % CI) of the association between miRNAs in extracellular vesicles and coronary heart disease history. All estimates were adjusted for age; body mass index (BMI); number of pack-years of smoking; total miRNA counts, and the number of red blood cells (RBCs), white blood cells (WBCs), and platelets. (DOCX 181 kb)

Additional file 7: Table S4. Odds ratios (95 % CI) of the association between miRNAs in extracellular vesicles and coronary heart disease history. (DOCX 15 kb)

## Abbreviations

AOD: Aerosol optical density
BH: Benjamini-Hochberg
BMI: body mass index
CHD: coronary heart disease
CI: confidence interval
CRP: C-reactive protein
CVD: cardiovascular disease
CXCL: C-X-C motif ligand
EDTA: Ethylenediaminetetraacetic acid
EVs: extracellular vesicles
FDR: false discovery rate
IL: interleukin
IPA: ingenuity pathway analysis
NAS: Normative Aging Study
NF-kB: nuclear factor-kappa-B
PBL: planetary boundary layer
PDGF-B: platelet-derived growth factor beta
PM: particulate matter
RBCs: red blood cells
RISC: RNA-induced silencing complex
SD: standard deviation
US EPA: United States environmental protection agency
VCAM-1: vascular adhesion molecule 1
WBCs: white blood cells
