# Supplementary figures and images for: Ambient particulate matter and microRNAs in extracellular vesicles: a pilot study of older individuals
Source: Part Fibre Toxicol. 2016 Mar 8;13:13. doi: 10.1186/s12989-016-0121-0 (PMC4782360; doi:10.1186/s12989-016-0121-0)

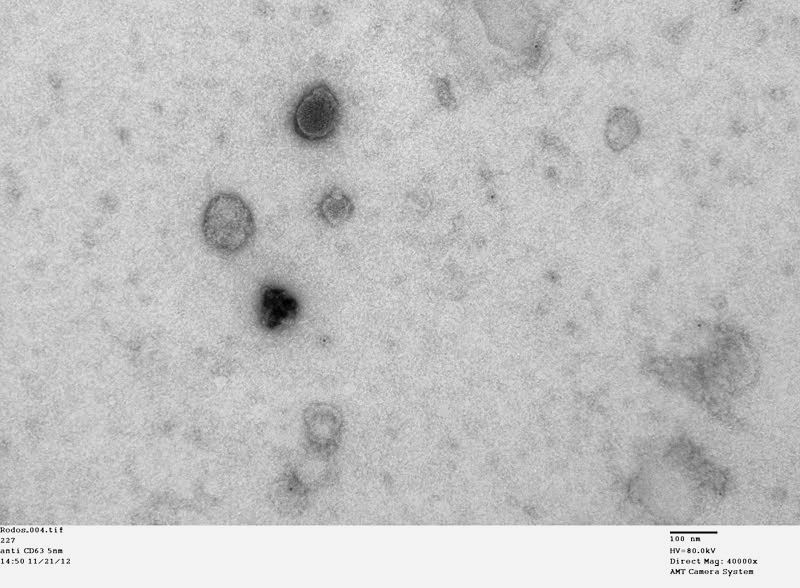


100 nm

**a**


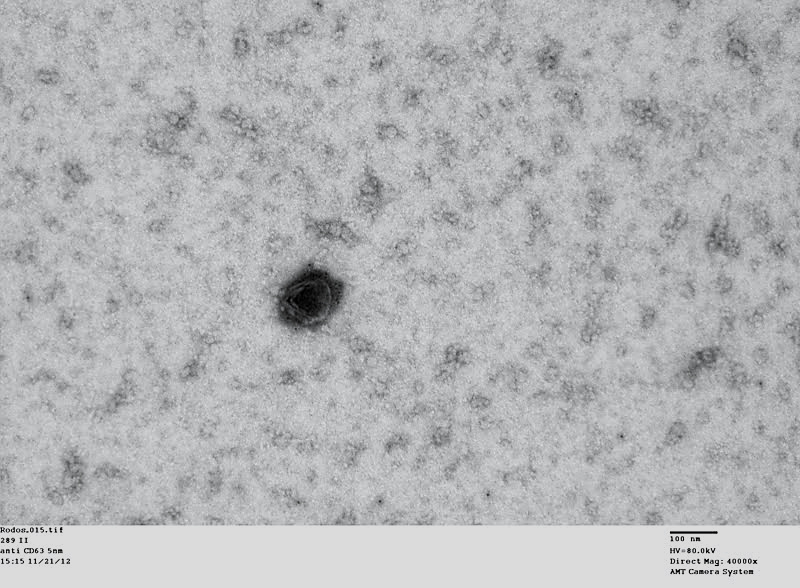


**b**

100 nm

**Anti-CD63**


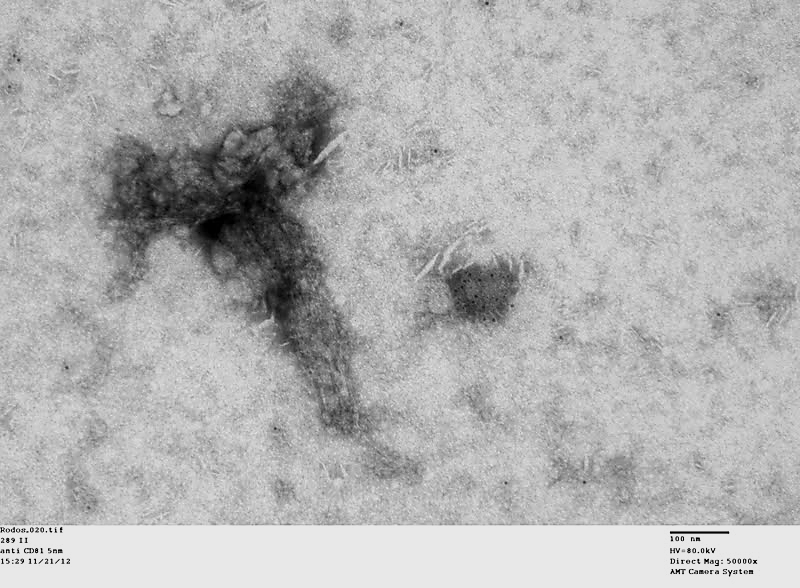


**c**

100 nm

**Anti-CD81**

Supplement: Supplementary file 1 — Morphological characterization of serum extracellular vesicles (EVs). Preparations of EVs were imaged by transmission electron microscopy (TEM). (a) non-labeled EVs, (b) EVs labeled with gold-conjugated anti-CD63 antibody, (c) EVs labeled with gold-conjugated anti-CD81 antibody. Images were taken by a JEOL 1200EX microscope coupled with an AMT 2 k CCD camera, at the Harvard Medical School Electron Microscopy Core. (DOCX 4627 kb) [file 12989_2016_121_MOESM1_ESM.docx]

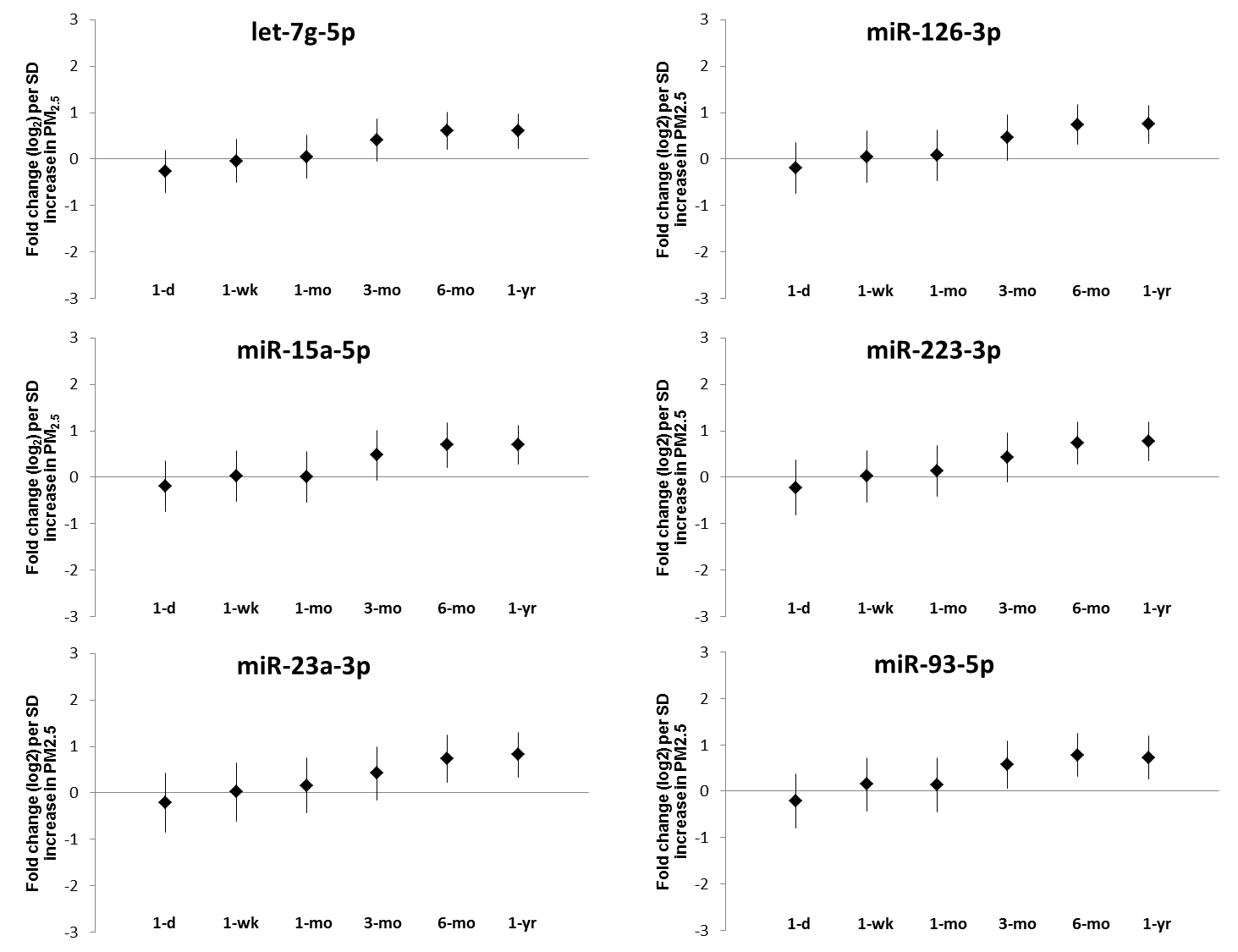

Supplement: Supplementary file 4 — Fold change (95 % CI) of selected miRNAs in extracellular vesicles over different time windows of ambient PM2.5 levels. Fold changes (95 % CI) for let-7 g-5p, miR-126-3p, miR-15a-5p, miR-223-3p, miR-23a-3p and miR-93-5p in response to ambient PM2.5 one-day, one-week, one-month, three-month, six-month, and one-year moving averages before blood sample collection, respectively; all estimates were adjusted for age; body mass index (BMI); number of pack-years of smoking; total miRNA counts, and the number of red blood cells (RBCs), white blood cells (WBCs), and platelets; SD indicates standard deviation. (DOCX 58 kb) [file 12989_2016_121_MOESM4_ESM.docx]

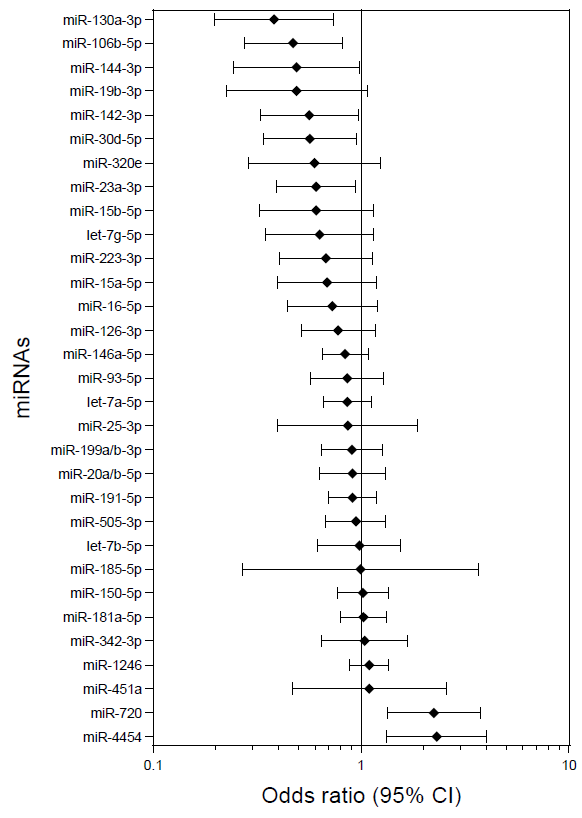

Supplement: Supplementary file 6 — Forest plot showing odds ratios (95 % CI) of the association between miRNAs in extracellular vesicles and coronary heart disease history. All estimates were adjusted for age; body mass index (BMI); number of pack-years of smoking; total miRNA counts, and the number of red blood cells (RBCs), white blood cells (WBCs), and platelets. (DOCX 181 kb) [file 12989_2016_121_MOESM6_ESM.docx]
