# Supplementary material for: Ambient particulate matter and microRNAs in extracellular vesicles: a pilot study of older individuals
Source: Part Fibre Toxicol. 2016 Mar 8;13:13. doi: 10.1186/s12989-016-0121-0 (PMC4782360; doi:10.1186/s12989-016-0121-0)
Supplement: Supplementary file 2 — Characteristics of the study participants at first examination (n = 22). (DOCX 18 kb) [file 12989_2016_121_MOESM2_ESM.docx]

| **Table S1:** Characteristics of study participants at first examination (n=22). | |
| --- | --- |
| Characteristic | Mean ± SD* |
| Age (years) | 75.0 ± 6.6 |
| BMI (kg/m^2^) | 26.8 ± 2.90 |
| Smoking status; n(%) |  |
| Never | 6 (27.2) |
| Current | 0 (0) |
| Former**^†^** | 16 (62.8) |
| Pack-years of smoking | 15.8 ± 15.2 |
| Race; n(%) |  |
| Non-hispanic white | 22 (100) |
| Coronary heart disease; n(%) | 7 (31.8) |
| Platelets (thousands/mm^3^ ) | 231.9 ± 52.8 |
| Red blood cells (thousands/mm^3^) | 4.7 ± 0.4 |
| White blood cells (thousands/mm^3^) | 6.5 ± 2.5 |
| *****Mean ± SD, unless otherwise specified.  **^†^**Former smokers quitted smoking at least 12 years prior to enrollment. | |
