# Supplementary material for: Ambient particulate matter and microRNAs in extracellular vesicles: a pilot study of older individuals
Source: Part Fibre Toxicol. 2016 Mar 8;13:13. doi: 10.1186/s12989-016-0121-0 (PMC4782360; doi:10.1186/s12989-016-0121-0)
Supplement: Supplementary file 5 — Associations between ambient PM2.5 moving average time windows and levels of miRNAs in extracellular vesicles. (DOCX 31 kb) [file 12989_2016_121_MOESM5_ESM.docx]

| **Table S3:** Associations between ambient PM_2.5_ moving average time windows and levels of miRNAs in extracellular vesicles. | | | | |
| --- | --- | --- | --- | --- |
|  | **miRNA** | **β coefficient^*^** | **SE** | **FDR adjusted *P* value** |
| **1-day** |  |  |  |  |
|  | miR-30d-5p | -0.56 | 0.17 | 0.03 |
|  | miR-25-3p | -0.30 | 0.14 | 0.16 |
|  | miR-342-3p | -0.44 | 0.22 | 0.20 |
|  | miR-106b-5p | -0.56 | 0.29 | 0.23 |
|  | miR-451a | -0.27 | 0.16 | 0.30 |
|  | miR-320e | -0.21 | 0.13 | 0.31 |
|  | miR-4454 | -0.33 | 0.23 | 0.37 |
|  | miR-19b-3p | -0.24 | 0.19 | 0.47 |
|  | let-7g-5p | -0.27 | 0.22 | 0.51 |
|  | miR-720 | -0.30 | 0.26 | 0.53 |
|  | miR-16-5p | -0.28 | 0.25 | 0.54 |
|  | miR-15b-5p | -0.25 | 0.26 | 0.64 |
|  | let-7b-5p | -0.34 | 0.37 | 0.64 |
|  | miR-505-3p | 0.26 | 0.32 | 0.74 |
|  | miR-223-3p | -0.23 | 0.29 | 0.74 |
|  | miR-185-5p | -0.08 | 0.12 | 0.78 |
|  | miR-93-5p | -0.21 | 0.28 | 0.78 |
|  | miR-23a-3p | -0.21 | 0.31 | 0.78 |
|  | miR-126-3p | -0.19 | 0.27 | 0.78 |
|  | miR-15a-5p | -0.19 | 0.27 | 0.79 |
|  | miR-150-5p | -0.20 | 0.32 | 0.79 |
|  | miR-130a-3p | -0.13 | 0.20 | 0.81 |
|  | miR-181a-5p | -0.23 | 0.41 | 0.81 |
|  | miR-199a/b-3p | -0.31 | 0.54 | 0.81 |
|  | miR-144-3p | -0.07 | 0.26 | 0.94 |
|  | miR-142-3p | -0.07 | 0.29 | 0.95 |
|  | let-7a-5p | 0.004 | 0.52 | 1.00 |
|  | miR-20a/b-5p | -0.03 | 0.40 | 1.00 |
|  | miR-146a-5p | 0.01 | 0.41 | 1.00 |
|  | miR-191-5p | 0.03 | 0.46 | 1.00 |
|  | miR-1246 | 0.07 | 0.76 | 1.00 |
| **1-week** |  |  |  |  |
|  | miR-451a | -0.33 | 0.15 | 0.16 |
|  | miR-25-3p | -0.21 | 0.14 | 0.37 |
|  | miR-720 | -0.33 | 0.25 | 0.44 |
|  | miR-4454 | -0.29 | 0.22 | 0.44 |
|  | miR-30d-5p | -0.23 | 0.20 | 0.53 |
|  | miR-191-5p | 0.44 | 0.45 | 0.64 |
|  | miR-20a/b-5p | 0.36 | 0.39 | 0.65 |
|  | miR-181a-5p | 0.33 | 0.41 | 0.72 |
|  | miR-150-5p | 0.22 | 0.32 | 0.78 |
|  | miR-185-5p | 0.08 | 0.12 | 0.78 |
|  | let-7a-5p | 0.36 | 0.51 | 0.79 |
|  | miR-146a-5p | 0.25 | 0.40 | 0.80 |
|  | miR-320e | -0.09 | 0.14 | 0.80 |
|  | miR-342-3p | -0.14 | 0.23 | 0.81 |
|  | miR-93-5p | 0.15 | 0.28 | 0.83 |
|  | miR-16-5p | -0.12 | 0.26 | 0.83 |
|  | miR-505-3p | 0.15 | 0.32 | 0.84 |
|  | miR-142-3p | 0.13 | 0.28 | 0.85 |
|  | miR-1246 | -0.28 | 0.73 | 0.90 |
|  | miR-106b-5p | -0.10 | 0.30 | 0.91 |
|  | miR-126-3p | 0.05 | 0.27 | 0.99 |
|  | let-7g-5p | -0.04 | 0.23 | 0.99 |
|  | miR-19b-3p | -0.03 | 0.20 | 1.00 |
|  | miR-144-3p | 0.0004 | 0.26 | 1.00 |
|  | miR-23a-3p | 0.03 | 0.30 | 1.00 |
|  | miR-15a-5p | 0.02 | 0.27 | 1.00 |
|  | miR-199a/b-3p | 0.04 | 0.53 | 1.00 |
|  | miR-15b-5p | -0.01 | 0.26 | 1.00 |
|  | miR-223-3p | 0.02 | 0.27 | 1.00 |
|  | let-7b-5p | -0.01 | 0.37 | 1.00 |
|  | miR-130a-3p | 0.01 | 0.20 | 1.00 |
| **1-month** |  |  |  |  |
|  | miR-451a | -0.24 | 0.15 | 0.30 |
|  | miR-19b-3p | 0.17 | 0.18 | 0.65 |
|  | miR-146a-5p | 0.27 | 0.40 | 0.78 |
|  | miR-150-5p | 0.23 | 0.32 | 0.78 |
|  | miR-1246 | -0.43 | 0.70 | 0.80 |
|  | miR-181a-5p | 0.26 | 0.41 | 0.80 |
|  | miR-191-5p | 0.26 | 0.45 | 0.82 |
|  | miR-23a-3p | 0.16 | 0.29 | 0.82 |
|  | miR-342-3p | -0.13 | 0.23 | 0.82 |
|  | miR-25-3p | -0.08 | 0.15 | 0.83 |
|  | miR-505-3p | -0.17 | 0.32 | 0.83 |
|  | miR-223-3p | 0.13 | 0.27 | 0.83 |
|  | let-7a-5p | 0.26 | 0.51 | 0.84 |
|  | miR-93-5p | 0.14 | 0.28 | 0.84 |
|  | miR-185-5p | 0.05 | 0.12 | 0.88 |
|  | miR-142-3p | 0.11 | 0.27 | 0.88 |
|  | miR-20a/b-5p | 0.13 | 0.39 | 0.91 |
|  | miR-126-3p | 0.09 | 0.27 | 0.92 |
|  | miR-16-5p | -0.07 | 0.26 | 0.94 |
|  | miR-106b-5p | -0.08 | 0.29 | 0.94 |
|  | let-7g-5p | 0.05 | 0.23 | 0.97 |
|  | miR-199a/b-3p | 0.11 | 0.52 | 0.98 |
|  | miR-144-3p | -0.04 | 0.25 | 0.99 |
|  | miR-130a-3p | -0.03 | 0.19 | 0.99 |
|  | miR-15b-5p | -0.02 | 0.24 | 1.00 |
|  | miR-15a-5p | 0.003 | 0.26 | 1.00 |
|  | miR-4454 | 0.01 | 0.22 | 1.00 |
|  | let-7b-5p | -0.05 | 0.38 | 1.00 |
|  | miR-720 | -0.01 | 0.25 | 1.00 |
|  | miR-30d-5p | 0.02 | 0.21 | 1.00 |
|  | miR-320e | 0.01 | 0.14 | 1.00 |
| **3-months** | |  |  |  |
|  | miR-19b-3p | 0.47 | 0.17 | 0.05 |
|  | miR-93-5p | 0.57 | 0.25 | 0.12 |
|  | miR-150-5p | 0.64 | 0.28 | 0.13 |
|  | miR-1246 | -1.56 | 0.73 | 0.17 |
|  | miR-142-3p | 0.50 | 0.26 | 0.22 |
|  | miR-106b-5p | 0.55 | 0.28 | 0.22 |
|  | miR-126-3p | 0.46 | 0.24 | 0.22 |
|  | let-7g-5p | 0.41 | 0.22 | 0.24 |
|  | let-7a-5p | 0.86 | 0.46 | 0.24 |
|  | miR-15a-5p | 0.48 | 0.26 | 0.25 |
|  | miR-15b-5p | 0.43 | 0.24 | 0.26 |
|  | miR-199a/b-3p | 0.81 | 0.46 | 0.27 |
|  | miR-20a/b-5p | 0.63 | 0.36 | 0.27 |
|  | miR-191-5p | 0.69 | 0.41 | 0.29 |
|  | miR-223-3p | 0.43 | 0.26 | 0.30 |
|  | miR-185-5p | 0.20 | 0.12 | 0.30 |
|  | let-7b-5p | 0.56 | 0.35 | 0.31 |
|  | miR-146a-5p | 0.56 | 0.37 | 0.36 |
|  | miR-23a-3p | 0.42 | 0.28 | 0.36 |
|  | miR-181a-5p | 0.55 | 0.38 | 0.37 |
|  | miR-130a-3p | 0.26 | 0.19 | 0.41 |
|  | miR-342-3p | 0.29 | 0.22 | 0.43 |
|  | miR-16-5p | 0.30 | 0.24 | 0.47 |
|  | miR-144-3p | 0.31 | 0.25 | 0.47 |
|  | miR-451a | -0.16 | 0.16 | 0.61 |
|  | miR-30d-5p | 0.18 | 0.19 | 0.65 |
|  | miR-25-3p | 0.12 | 0.14 | 0.70 |
|  | miR-720 | -0.18 | 0.25 | 0.78 |
|  | miR-4454 | -0.16 | 0.22 | 0.80 |
|  | miR-505-3p | 0.11 | 0.31 | 0.91 |
|  | miR-320e | -0.02 | 0.14 | 0.99 |
| **6-months** | |  |  |  |
|  | miR-126-3p | 0.74 | 0.21 | 0.02 |
|  | miR-19b-3p | 0.52 | 0.15 | 0.02 |
|  | miR-93-5p | 0.78 | 0.22 | 0.02 |
|  | miR-223-3p | 0.74 | 0.22 | 0.02 |
|  | miR-142-3p | 0.81 | 0.21 | 0.03 |
|  | let-7a-5p | 1.41 | 0.41 | 0.03 |
|  | miR-1246 | -2.04 | 0.60 | 0.03 |
|  | miR-199a/b-3p | 1.45 | 0.39 | 0.03 |
|  | let-7g-5p | 0.61 | 0.20 | 0.04 |
|  | miR-191-5p | 1.13 | 0.37 | 0.04 |
|  | miR-15a-5p | 0.69 | 0.23 | 0.04 |
|  | miR-23a-3p | 0.74 | 0.25 | 0.04 |
|  | miR-15b-5p | 0.60 | 0.21 | 0.06 |
|  | miR-20a/b-5p | 0.91 | 0.33 | 0.06 |
|  | miR-144-3p | 0.57 | 0.22 | 0.07 |
|  | let-7b-5p | 0.83 | 0.33 | 0.08 |
|  | miR-150-5p | 0.96 | 0.24 | 0.09 |
|  | miR-146a-5p | 0.85 | 0.35 | 0.10 |
|  | miR-130a-3p | 0.40 | 0.17 | 0.12 |
|  | miR-181a-5p | 0.83 | 0.36 | 0.13 |
|  | miR-505-3p | 0.65 | 0.28 | 0.13 |
|  | miR-106b-5p | 0.54 | 0.27 | 0.19 |
|  | miR-185-5p | 0.19 | 0.11 | 0.29 |
|  | miR-451a | -0.25 | 0.15 | 0.30 |
|  | miR-342-3p | 0.34 | 0.21 | 0.31 |
|  | miR-16-5p | 0.35 | 0.23 | 0.35 |
|  | miR-4454 | -0.21 | 0.21 | 0.63 |
|  | miR-25-3p | 0.14 | 0.14 | 0.63 |
|  | miR-720 | -0.21 | 0.25 | 0.71 |
|  | miR-30d-5p | 0.08 | 0.20 | 0.88 |
|  | miR-320e | -0.05 | 0.14 | 0.90 |
| **1-year** |  |  |  |  |
|  | miR-23a-3p | 0.83 | 0.23 | 0.02 |
|  | miR-150-5p | 0.90 | 0.24 | 0.02 |
|  | miR-15a-5p | 0.70 | 0.21 | 0.02 |
|  | miR-191-5p | 1.20 | 0.35 | 0.02 |
|  | let-7a-5p | 1.42 | 0.39 | 0.02 |
|  | let-7g-5p | 0.60 | 0.18 | 0.03 |
|  | miR-126-3p | 0.75 | 0.20 | 0.03 |
|  | miR-223-3p | 0.77 | 0.20 | 0.03 |
|  | miR-93-5p | 0.72 | 0.22 | 0.03 |
|  | miR-505-3p | 0.83 | 0.26 | 0.03 |
|  | miR-199a/b-3p | 1.49 | 0.38 | 0.03 |
|  | miR-130a-3p | 0.51 | 0.16 | 0.03 |
|  | miR-146a-5p | 0.98 | 0.33 | 0.04 |
|  | miR-142-3p | 0.86 | 0.22 | 0.05 |
|  | miR-1246 | -1.77 | 0.63 | 0.06 |
|  | let-7b-5p | 0.87 | 0.32 | 0.06 |
|  | miR-20a/b-5p | 0.88 | 0.33 | 0.06 |
|  | miR-144-3p | 0.60 | 0.22 | 0.06 |
|  | miR-15b-5p | 0.59 | 0.22 | 0.07 |
|  | miR-181a-5p | 0.86 | 0.35 | 0.10 |
|  | miR-19b-3p | 0.37 | 0.17 | 0.16 |
|  | miR-106b-5p | 0.50 | 0.27 | 0.24 |
|  | miR-451a | -0.27 | 0.15 | 0.26 |
|  | miR-185-5p | 0.18 | 0.11 | 0.30 |
|  | miR-342-3p | 0.28 | 0.21 | 0.43 |
|  | miR-16-5p | 0.27 | 0.23 | 0.53 |
|  | miR-4454 | -0.26 | 0.22 | 0.53 |
|  | miR-25-3p | 0.15 | 0.14 | 0.53 |
|  | miR-720 | -0.28 | 0.26 | 0.55 |
|  | miR-30d-5p | 0.05 | 0.19 | 0.95 |
|  | miR-320e | 0.004 | 0.14 | 1.00 |
| ^*^Regression coefficient estimating the effect on ex-miRNA levels (log_2_) for every SD increase in PM_2.5_ levels; models adjusted for age, BMI, pack-years of smoking, RBC, WBC and PLT. | | | | |
|  |  |  |  |  |
|  |  |  |  |  |
