# Supplementary material for: Ambient particulate matter and microRNAs in extracellular vesicles: a pilot study of older individuals
Source: Part Fibre Toxicol. 2016 Mar 8;13:13. doi: 10.1186/s12989-016-0121-0 (PMC4782360; doi:10.1186/s12989-016-0121-0)
Supplement: Supplementary file 7 — Odds ratios (95 % CI) of the association between miRNAs in extracellular vesicles and coronary heart disease history. (DOCX 15 kb) [file 12989_2016_121_MOESM7_ESM.docx]

| **Table S4:** Odds ratios (95% CI) of the association between miRNAs in extracellular vesicles and coronary heart disease history. | | | |
| --- | --- | --- | --- |
|  |  |  |  |
| **miRNA** | **OR^†^** | **95% CI** | **FDR adjusted  *P value*** |
| miR-130a-3p | 0.38 | (0.20, 0.74) | 0.04 |
| miR-4454 | 2.32 | (1.33, 4.04) | 0.05 |
| miR-106b-5p | 0.47 | (0.27, 0.81) | 0.05 |
| miR-720 | 2.24 | (1.34, 3.75) | 0.07 |
| miR-23a-3p | 0.61 | (0.39, 0.94) | 0.16 |
| miR-30d-5p | 0.57 | (0.34, 0.95) | 0.16 |
| miR-142-3p | 0.56 | (0.33, 0.97) | 0.17 |
| miR-144-3p | 0.49 | (0.24, 0.98) | 0.17 |
| miR-19b-3p | 0.49 | (0.22, 1.07) | 0.25 |
| miR-223-3p | 0.68 | (0.40, 1.14) | 0.36 |
| miR-15a-5p | 0.69 | (0.40, 1.19) | 0.37 |
| miR-146a-5p | 0.84 | (0.65, 1.08) | 0.38 |
| let-7g-5p | 0.63 | (0.35, 1.15) | 0.38 |
| miR-15b-5p | 0.61 | (0.32, 1.15) | 0.39 |
| miR-320e | 0.60 | (0.29, 1.24) | 0.40 |
| miR-16-5p | 0.73 | (0.44, 1.20) | 0.41 |
| miR-126-3p | 0.78 | (0.51, 1.17) | 0.42 |
| let-7a-5p | 0.86 | (0.65, 1.13) | 0.47 |
| miR-1246 | 1.09 | (0.88, 1.36) | 0.68 |
| miR-93-5p | 0.86 | (0.57, 1.28) | 0.70 |
| miR-191-5p | 0.91 | (0.70, 1.19) | 0.74 |
| miR-199a/b-3p | 0.90 | (0.64, 1.27) | 0.79 |
| miR-20a/b-5p | 0.91 | (0.63, 1.32) | 0.84 |
| miR-505-3p | 0.94 | (0.68, 1.31) | 0.91 |
| miR-25-3p | 0.86 | (0.40, 1.88) | 0.92 |
| miR-150-5p | 1.02 | (0.77, 1.36) | 0.94 |
| let-7b-5p | 0.98 | (0.62, 1.56) | 0.96 |
| miR-342-3p | 1.04 | (0.65, 1.66) | 0.97 |
| miR-181a-5p | 1.02 | (0.79, 1.32) | 0.98 |
| miR-451a | 1.10 | (0.47, 2.57) | 0.99 |
| miR-185-5p | 1.00 | (0.27, 3.68) | 1.00 |
| †All models were adjusted for age, body mass index (BMI), pack-years of smoking, total miRNA counts, and the number of red blood cells (RBCs), white blood cells (WBCs), and platelets. | | | |
|  |  |  |  |
|  |  |  |  |
